# Challenges and recommendations for collecting and quantifying implementation costs in practice: a qualitative interview study

**DOI:** 10.1186/s43058-024-00648-y

**Published:** 2024-10-11

**Authors:** Thomasina Donovan, Hannah E. Carter, Steven M. McPhail, Bridget Abell

**Affiliations:** 1 Australian Centre for Health Services Innovation and Centre for Healthcare Transformation, School of Public Health and Social Work, Faculty of Health, Queensland University of Technology, Brisbane, Qld Australia; 2 Digital Health and Informatics, Metro South Health, Brisbane, Qld Australia

**Keywords:** Implementation costs, Implementation science, Health economics, Digital health

## Abstract

**Background:**

The cost of implementation is typically not accounted for in published economic evaluations, which determine the relative value for money of health innovations and are important for allocating scarce resources. Despite key papers outlining relevant implementation costs, they continue to be under reported in the literature and often not considered in practice. This study sought to understand and outline current practices for capturing the costs associated with implementation efforts, with examples from the digital health setting.

**Methods:**

A qualitative study of semi-structured interviews with purposefully sampled experts in implementation science, health economics and/or digital health was conducted. The interview guide was informed by a literature review and was pilot tested. Interviews were digitally recorded and transcribed. A hybrid inductive/deductive framework analysis was conducted using thematic analysis to elicit key concepts related to the research question.

**Results:**

Interviews were conducted with sixteen participants with specialist expertise in implementation science (*n* = 8), health economics (*n* = 6), and/or digital health (*n* = 8). Five participants were experienced in more than one field. Four key themes were elicited from the data: difficulty identifying and collecting implementation cost data; variation in approaches for collecting implementation cost data; the value of implementation costs; and collaboration enables implementation costing. Broadly, while interviewees recognised implementation costs as important, only some costs were considered in practice likely due to the perceived ill-defined boundaries and inconsistencies in terminology. A variety of methods were used to collect and estimate implementation costs; the most frequent approach was staff time tracking. Multidisciplinary collaboration facilitated this process, but the burden of collecting the necessary data was also highlighted.

**Conclusions:**

In current practice, standardised methods are not commonly used for data collection or estimation of implementation costs. Improved data collection through standardised practices may support greater transparency and confidence in implementation cost estimates. Although participants had industry exposure, most were also academic researchers and findings may not be representative of non-academic industry settings.

**Supplementary Information:**

The online version contains supplementary material available at 10.1186/s43058-024-00648-y.

Contribution to the literature

1. In practice, implementation cost estimates are influenced by the wide variation in methodological approaches, the burdensome nature of data collection, and the availability of data collection tools.
2. Standardised tools to collect and estimate implementation costs may promote transparency, support cross study comparison and increase confidence in implementation cost estimates.
3. Costing implementation was hindered by the perceived ill-defined boundaries of implementation and inconsistencies in terminology used across different academic backgrounds.
4. The collection and estimation of implementation cost data is likely to be facilitated through the identification and categorisation of project activities, to a level of detail that is appropriate for the project.

## Introduction

It is essential to estimate the cost of healthcare models, services, or interventions to support the appropriate allocation of healthcare-related resources [1]. Economic evaluations are employed to conduct this work but often exclude the costs associated with implementation strategies [2, 3]. Implementation strategies require additional resourcing to facilitate the adoption of healthcare interventions. Under resourcing can lead to failed implementation, while over resourcing can lead to inefficient use of scarce healthcare resources [4]. One challenge when applying economic evaluations to implementation strategies relates to obtaining accurate implementation cost estimates [5, 6], in an efficient way [7]. Despite key papers outlining relevant costs and sources for cost data [8–10], implementation costs continue to be under reported in the literature [11] and often not considered in practice [12]. Tools to assist the collection of appropriate implementation cost data may improve reporting, and in turn the impact of economic evaluations in implementation science [4, 5]. However, to date, research suggests that it’s been difficult to balance the ability for a tool to be both comprehensive and practical, while also minimising the need for prior implementation science knowledge [13, 14].

Implementation costing is a transdisciplinary issue requiring expertise from fields including implementation science and health economics. Collaboration between fields may help to overcome previously documented difficulties related to tracking implementation costs, variation in resource needs across implementation stages, and reluctance of sharing financial information, which has contributed to the lack of economic evaluations in implementation science studies [15]. Implementation scientists and health economists share motivation to collaborate to improve methodological rigor and real-world impact, however, collaboration is currently underutilised [15, 16].

Digital health is one field where there are increasing attempts to measure the costs and cost-effectiveness of their digital interventions [17]. Digital health innovations include tele-health, electronic reminder systems, artificial intelligence (AI) and machine learning, and may provide better value care by reducing human error, improving clinical outcomes, facilitating care coordination, improving practice efficiency, or tracking data over time [18]. Improvements in efficiency, time or effort may also translate into cost savings [19, 20]. However, the costs associated with implementation strategies for digital health innovations have been under reported in the literature, and often excluded from economic evaluations [11, 21, 22]. For example, a 2023 review identified only nine studies to report the costs associated with the implementation of clinical decision support systems, most of which did not contain enough information to discern data collection practices [23]. Specific challenges to costing implementation in the digital health setting include inconsistencies in the concept of implementation costs and a lack of methodological guidance suited to the digital health context [23].

The aim of this study was to understand and outline current practices for capturing the costs associated with implementation efforts, with examples from the digital health setting. It is intended that the findings will contribute to ongoing research in this field to establish effective and efficient data collection practices for estimating costs associated with implementation efforts.

## Methods

### Study design

We conducted a qualitative study using semi-structured interviews to document how the implementation of digital health innovations has been costed in hospital settings. The study sought to understand processes, experiences, opinions, and feelings attributed to this phenomenon. To achieve this we adopted a qualitative exploratory and descriptive approach using a hybrid inductive/deductive framework analysis [24]. This approach is consistent with that recommended for healthcare research and multidisciplinary teams [25]. The Consolidated Criteria for Reporting Qualitative Research Checklist (COREQ) was used for transparent and complete reporting of methods (see Additional file 1) [26]. Ethical approval was obtained from Metro South Human Research Ethics Committee (HREC/2022/QMS/81677).

### Study participants and recruitment

A purposive sampling approach was used to recruit stakeholders from the academic, government, clinical or health service sectors who had experience working in the fields of implementation science, health economics and/or digital health. Potential participants were identified through existing collaborative research networks, publicly available hospital and university staff directories, and key academic publications in the fields of implementation science and health economics. We examined publicly available biographies of potential participants, including academic staff biographies, to confirm experience in relevant fields. Researcher TD emailed an invitation to participate and study information sheet. Interviews were arranged for those who expressed interest in participating and provided consent. While recruitment mostly occurred at the local and national level, international participants were also eligible for inclusion. An estimated sample size of 5 to 25 participants was preliminarily established to provide depth in data collection, with the final sample size being determined based on both pragmatic considerations and thematic saturation of data [27]. Thematic saturation of data was present when no new themes were noted after four successive interviews (the stopping criterion) [28]. A total of sixteen participants were interviewed.

### Data collection

Semi-structured interviews were conducted from May to November 2022, using an interview guide (Additional file 2). The interview guide was informed by a literature search and internally tested through discussion and mock interviewing (TD, BA, and HC) [29]. It was then piloted in interviews with a sample group of implementation scientists and health economists with experience in digital health (*n* = 4), representative of the study population. The interview guide was further refined after the pilot. Final topics included how participants defined implementation costs; how they differ from intervention costs; why and how implementation costs are recorded; and the importance of doing so. The pilot data was used in the final analysis.

Depending on the participant’s preference and proximity to the research team, individual interviews were either conducted in-person or virtually, using the Zoom videoconferencing platform. One researcher (TD) conducted all interviews which lasted between 30 and 45 min and were audio recorded with consent. On occasion a second researcher (MF) was present in the interviews. Both TD and MF were female PhD students, with a research focus on implementation science, health economics and digital health. They received training in semi-structured interviewing and guidance from BA, an implementation scientist who has extensive experience in qualitative research. The interviewer/s were not known to participants prior to this study, however the wider research team (HC, BA, SM) was known to some participants. Participants did not have the opportunity to review, comment on or change their answers after the interview had taken place. No repeat interviews were conducted. Research notes and reflective memos were recorded by TD before and after each interview, and throughout data collection and analysis.

Data collection and data analysis were iterative. Initially six interviews were conducted, analysed and themes identified. Then a further six interviews were conducted and analysed. This iterative process continued until the research team agreed that both data saturation and inductive thematic saturation had been achieved [30]. The point of data saturation was defined when four successive interviews were conducted and no new themes emerged, this was the stopping criterion [28].

### Data analysis

We followed the procedure described by Gale and colleagues for using framework analysis in multidisciplinary health research teams [25]. Table 1 outlines how we performed Gale’s seven stages of framework analysis in the context of this study [24]. In addition, the costs and resources associated with implementation strategies described by participants were mapped to the Expert Recommendations for Implementing Change (ERIC) framework [31].

**Table 1:** Framework data analysis

Framework analysis stage	Method
Stage 1: Transcription	Audio recordings were transcribed using Microsoft Office Word’s transcribe functionality. All transcripts were checked for accuracy by TD who listened to the recording and corrected the transcript in real-time. Transcripts were de-identified.
Stage 2: Familiarisation with the interview	The research team became familiar with the data by thoroughly reading through transcripts and listening to audio recordings.
Stage 3: Coding	Three researchers (TD, BA, HC) independently semantically coded the same transcript. Line by line inductive coding was conducted to authentically reflect the participant’s voice.
Stage 4: Developing a working analytical framework	The research team then met to compare and discuss the identified codes. Codes were then deductively allocated to themes and sub-themes established a priori from the interview guide, reflecting a deductive framework approach. An initial framework was developed from this set of agreed codes An additional two transcripts were then coded by the research team using the initial coding framework. The research team met again to discuss and revise the framework.
Stage 5: Applying the analytical framework	The revised coding framework was systematically applied to all transcripts by one researcher (TD) using NVivo. Any new codes or themes falling outside of the framework were recorded and discussed with the research team. After all transcripts were coded the framework was refined further in consultation with the entire research team. The refined framework consisted of themes, subthemes, and codes. Codes described distinct ideas. Similar codes were grouped into subthemes that described the code’s relationship. Subthemes were allocated within higher level themes that were developed a priori from the interview guide.
Stage 6: Charting data into the framework matrix	While we did not use traditional data charting methods and matrices, this step of the analysis still involved thematic summarisation of data across all interviews.
Stage 7: Interpreting the data	The data within the finalised framework was thematically analysed to identify connections between and within codes and themes. Themes were discussed and agreed on within the entire research team.

The research team that analysed the data included an implementation scientist (BA) and a health economist (HC) which reflected the participant population and allowed discipline nuances to be captured. Regular meetings were held during data analysis to discuss codes, subthemes, and themes to ensure coherency and consistency with the data to maximise rigour. Research notes were not subjected to thematic analysis but assisted in understanding and developing codes, subthemes, and themes [32]. Data analysis was conducted using NVivo software (release 1.6.1).

## Results

### Participant characteristics

**Fig. 1:**
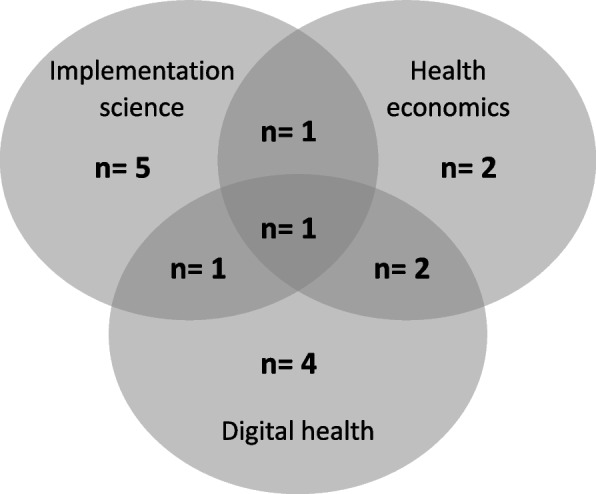
Participants’ experience in implementation science, health economics and digital health

Sixty-two professionals with experience in implementation science (IS), health economics (HE) or digital health (DH) were invited to participate in the study. Interviews were conducted with sixteen consenting participants: five implementation scientists, two health economists, four digital health specialists and five with experience across more than one of these fields (Fig. 1). Participants worked across a range of healthcare disciplines, clinical areas and settings including nursing, surgery, maternal health, nutrition and dietetics, pharmacy, heart disease, lung cancer, clinical excellence, information systems, and digital health including telehealth and AI. Most participants were worked in academia (*n* = 14) and were located in the same geographical region as the research team (*n* = 9) (Table 2).

**Table 2:** Participant characteristics

Characteristic	Count
**Location**	
National (Australia)	14
*Queensland (local)*	*9*
*New South Wales*	*2*
*Victoria*	*2*
*South Australia*	*1*
International	2
*United States*	*1*
*Canada*	*1*
**Industry**	
Academic and Healthcare	7
Academic only	7
Government	1
Consultancy	1

### Themes

**Fig. 2:**
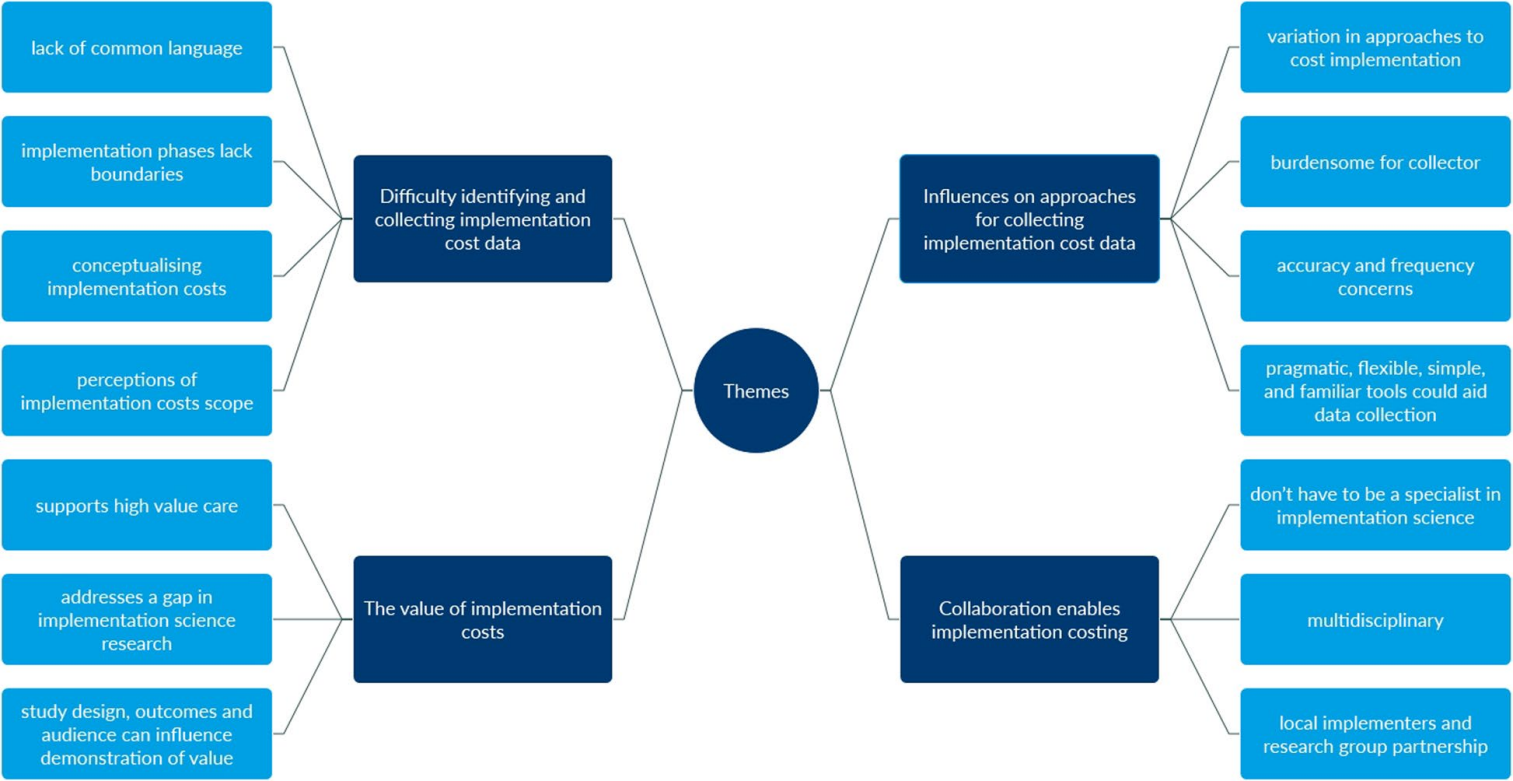
Thematic analysis coding tree

Four major themes, each containing three or four subthemes, were derived from the data (as seen in Fig. 2) and are explained in more detail below and summarised in Table 3. Three deductive themes of *difficulty identifying and collecting implementation cost data*, *influences on approaches for collecting implementation cost data*, and *the value of implementation costs* were developed as a priori from the interview guide based on the research question. One theme of *collaboration enables implementation costing* occurred inductively from the data.

**Table 3:** Themes, subthemes and quotes. (NVivo File V6.2)

Theme and subtheme	Quote
1. Difficulty identifying and collecting implementation cost data	
1.1 Lack of common language	(a) “Even within digital health, you get people with clinical backgrounds, you get people from technical backgrounds and they talk a different language sometimes. But digital health sort of brings that together somewhat. Then implementation science framework background is different again to health economists. Yeah, I agree that common language is really important. And that's why we need to be clear about this sort of stuff.” [IS/HE/DH] (b) “Yep, deployed/implementation, so we use them interchangeably.” [DH]
1.2 Implementation phases lack boundaries	(a) “The line as to when that becomes implementation, when that starts to be implementation is probably a bit murky.” [HE/DH] (b) “But it's still not to me, everything's implementation. No matter where you are in the innovation cycle like it's it's it's-Innovation cycle is only a path to implementation so that you gotta think.” [IS/DH] (c) “*chuckle* I mean, it depends what you mean when you say implementation. Do you mean like the planning of it as well? Like from the start of the project 'cause there's like so many costs that I don’t wanna go into they're gonna confuse things when you're talking about like developing an implementation plan or like a protocol type thing.” [IS/DH] (d) “Well, that's the other thing. When you take the implementation costs, you know, do you include your evaluation costs as well? Because that's, you know another thing. And if you're talking about something realistic then you should be including evaluation costs because every intervention in theory, if you're doing evidence based practice should have an evaluation and that should be costed whatever that looks like. So you've got, and that's another part of your implementation. Whether that's sort of pre or post or during or yeah." [HE/DH] (e) “I think one of the things is you should separate out the research cost, 'cause I didn't want to include those versus the cost of actually changing practice.” [IS/HE]
1.3 Conceptualising implementation costs	(a) “So I guess 2 streams of costs, but one short term project costs and the other ones just delivery.” [IS] (b) “… so your intervention will be- have some sort of ongoing cost. Most of them do, whether it's staff or like your ongoing digital support costs. Whereas your implementation ones should have a- I would normally say should have a reasonable time frame associated with them. They're not forever because eventually something should become part of standard practice”. [HE] (c) “And so the implementation costs would be if you did need a champion who was an extra staff member.” [IS/HE] (d) “But then with the implementation we would probably have an ongoing nurses training and things like that each year.” [HE] (e) “And so we could probably have looked at costs both in terms of project time for the project officer to manage the project.” [IS] (f) “I mean the major cost there probably is a person who is then responsible for implementing.” [IS/HE] (g) “It’s very much around an understanding the scale of the problem and trying to identify where data was.” [DH] (h) “There's a whole piece around making sure that the context of the clinical setting is appropriately considered.” [HE/DH]
1.4 Perceptions of implementation costs scope	(a) “I think there's clearly a distinction between implementation costs and the costs that we're trying to intervent.” [HE] (b) “… we collected all of that information as an implementation cost. And that is actually quite distinct from the intervention itself. But you know there's definitely overlap because the intervention did involve education, so the intervention itself was an educational program. Whereas the implementation was more the sort of regular, the regular contacts.” [IS/DH] (c) “The videos didn't cost us anything 'cause we have in-house marketing teams, so it didn't cost, we didn’t get a bill for that.” [DH] (d) “And the problem is, if you're talking implementation time, your staff aren't going to differentiate between implementation as such like and just time doing their job and everything.” [HE/DH] (e) “You know, like if I think about people who are really and successful at introducing new ideas, then it is the softer skills they've got around knowing how to get difficult people on board and build the types of relationships. And yeah, I'm not sure that in terms of the costs that that's easy to capture.” [IS]
2. Influences on approaches for collecting implementation cost data	
2.1 Variation in approaches to cost implementation	(a) “So it sort of goes from easy if you can just … have somebody pull the data all the way through to sort of grinding to get the data yourself.” [HE/DH] (b) “So every time one of those outreach workers did anything we asked them to complete a form. So…whenever they talked to the women we wanted, like the minutes and sort of what was done in the implementation activities.” [HE] (c) “So I'm probably a bad interviewer here when I say expert opinion. And if those unavailable, they use next best estimates. So again expert opinion and trying to get as close to the mark as possible.” [HE/DH] (d) “We knew how much money was available, so we fitted into that budget.” [DH]
2.2 Burdensome for collector	(a) “*sigh* staff time tracking is difficult because it requires your staff members to be on board.” [HE/DH] (b) “If you ask someone else to do that on top of the existing work, and there’s no incentive for them to do it, then that’s going to seem like a huge task. But if it was someone who, as part of the project implementation they were expected to record who was at the meeting and people wrote down their job classification, that wouldn’t be unreasonable.” [HE/DH]
2.3 Accuracy and frequency concerns	(a) “It was such a burden to capture that level of precision.” [HE] (b) “We like breakdown every single task that they're gonna do in the study. It's not always super accurate, but you know we get as close as we can.” [IS/DH] (c) “It's often estimated at the start but it may not be necessarily validated by concurrent recording. You know we could say we trained a 100 people and we could estimate what that costs. I mean that could happen, but not actually probably does that.” [IS/HE/DH]
2.4 Pragmatic, flexible, simple, and familiar tools could aid data collection	(a) “If we're listening to what people want on the ground, they want tools. And they want really practical tools and they want tools that directly help them solve the problems that they're creating.” [HE/DH] (b) “It would have to be in a pragmatic way that that could be done, ideally using stuff that has to be recorded for other purposes anyway.” [IS/HE/DH] (c) “So you also have to have your categories really clear for what you want them to be tracking. And you can't have too many, otherwise they won't pay any attention or use any of them. So time tracking is like amazing in theory and really difficult in practice.” [HE/DH] (d) “a simple tool like simple way of people who are implementing innovations locally. Because you know we got a lot of research stuff out there but and it helps and supports researchers, but it doesn't really as I said, when a lot of these innovations don't make it to research.” [IS/DH] (e) “Think I know what would make it easier. Think of it, if it's electronic, it's easier.” [IS] (f) “Easy for me, yeah. We use, here at [place of work], we use the Microsoft suite of applications a lot. So there's a lot of digital record keeping for project managers. So we use OneNote and MS Teams and teams chats. Connecting teams in one note so you can record meeting attendance and how long the meeting went for.” [IS/HE/DH] (g) “So you know, uh, educating staff, holding meetings with the senior leaders, running group sessions, and doing some auditing. And so that was all just set up in red cap. And then obviously there was an other where they could add in things maybe that we hadn't anticipated.” [IS]
3. The value of implementation costs	
3.1 Supports high value care	(a) “You have to show some benefit to the system. It either has to be beneficial for outcomes, clinical, because that's cost saving. Or beneficial for patient experience, because that's really important to health systems and should be. Or financially beneficial. So it has to fulfil at least one of those 3 criteria I think if you're gonna do a service redesign.” [DH] (b) “I really want to produce a really informative report that outlines what it would take to implement this digital health initiative statewide.” [IS/HE/DH] (c) “I've been involved with projects that get up one of two ways. They either get a small pilot grant to run a pilot, in which case you need to outline a budget and how you're going to do something. Or through a business case…If it goes on to be a permanent service, that you know you again, you need a budget to justify how you're going to do it. Yeah, because you know the execs are not going to support something that hasn't got funding behind it.”[HE/DH]
3.2 Addresses a gap in implementation science research	(a) “Because I think it's been such a massive gap in implementation science.” [IS] (b) “I mean honestly, I think no one tracking it. I think no one is asking those questions. I think that… yeah. And I think it's a huge game changer.” [IS] (c) “So I won't think about the process of the health system of setting up a new service or anything like that. Unless the funder wants me to consider those attributes. *In your experience, have you found funders or whoever is wanting this evaluation-?* Not yet, no. I don't think I have seen those that have implementation cost as you call them in any of my modules. But this should happen I think.” [HE]
3.3 Study design, outcomes and audience can influence demonstration of value	(a) “…it didn't meet the primary objective and didn't end up going down that pathway of analysing the data further.” [DH] (b) “No, I haven't [evaluated implementation costs]… We did write in a health economist, but we just didn't get the funding.” [IS] (c) “And deciding whether or not they're [implementation costs] actually relevant to the end user of your evaluation. It really depends who your evaluation is for.” [HE/DH] (d) “I mean because when we've done evaluations in the past nobody ever asked for those.” [DH]
4. Collaboration enables implementation costing	
4.1 Don’t have to be a specialist in implementation science	(a) “Yes, in that we have somebody on our team who has implementation experience. But honestly I think we've been doing implementation work, so I work within a team of 6 academics and we all have different clinical backgrounds and different sort of subspecialties with how we- when it comes to evaluation. So we've got a psychologist who is our qual-expert, but we've got, you know, I'm probably the main point person, and then we do have somebody with an implementation science background, but as yet we haven't utilized that largely in our in our studies.” [HE/DH]
4.2 Multidisciplinary	(a) “So, so now my practice had my research practice in a sense, has changed where the health economist is there right from the beginning. And that's been a learning experience over the last 6–6 or so years. But you do that negotiation and get somebody in your team and you build up those relationships with the health economist as an important part of implementation research.” [IS] (b) “So all of that was planned out ahead of time with a health economist and getting advice from him in the design of the project.” [IS] (c) “But in a study where we have like an actual economic component where we're doing some kind of economic analysis, I leave all of that to the health economists. I know my place hahah.” [IS/DH]
4.3 Local implementers and research group partnership	(a) “Yes and so I think this is where it's really important for your evaluation staff or your health economists or whatever you want to call it, and you really need everybody to be talking. Like and you need clinicians involved because otherwise the encounters like myself can go we'll do time based management measurement and everybody goes, oh yes, that sounds great and then you go to the clinicians they go what?” [HE/DH]

*IS* Implementation scientist, *HE* Health economist, *DH* Digital health expert

#### Difficulty identifying and collecting implementation cost data

Across the fields of implementation science, health economics and digital health, terminology differed and caused confusion, specifically when identifying appropriate implementation cost data. In digital health, *“they [digital health solutions] typically get kind of deployed, as in I want to put this system in and then I want to use it right? So that that business of putting it in and using it is what we describe as implementation. …deployed/implementation, we use them interchangeably” [DH]*. In implementation science, the process of implementation was considered broader and included considerations of context at the patient, provider, system and/or policy levels. A common language across fields was lacking, yet was perceived to be important when costing implementation.

**Fig. 3:**
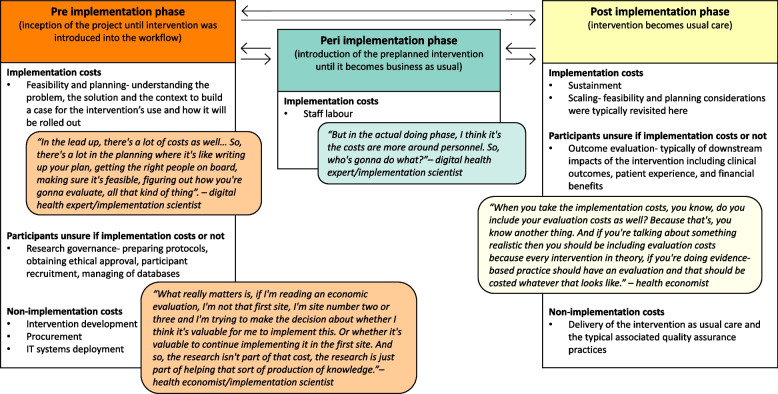
Implementation phases and associated activities

The boundaries of implementation were difficult to delineate for participants which added to the difficulty of identifying appropriate implementation costs (Table 3: quote 1.2.(a)). For example, some participants were unsure when to start costing implementation (e.g., whether to include project planning activities) and when to end costing implementation (e.g., whether to include evaluation activities) (Table 3: quote 1.2.(b)-(d)). Although the bounds of implementation were unclear to the participants, certain activities and associated costs (both implementation and non-implementation costs) were often discussed in phases. The phases were not linear but were discussed in a logical order, from pre to post, while acknowledging the cyclical nature of implementation projects. To better understand the presence and types of costs across the implementation continuum we arranged these activities and associated costs into three phases defined as pre-implementation, peri-implementation or post-implementation, as seen in Fig. 3.

There was a wide variation of how participants conceptualized implementation costs contributing to difficulties identifying implementation cost data. Some participants believed, *“intervention costs would include how the intervention was implemented” [HE]* while others felt it was mainly implementation strategies, *“for us, the implementation cost was largely the facilitation” [IS]*. Other ways implementation costs were described included short-term costs, ‘extra’ costs, specific personnel, and pieces of work (Table 3: quote 1.3.(a)-(g)).

Participants found some implementation costs and intervention costs easier to identify than others (Table 3: quote 1.4.(a)/(b)). Implementation strategies were the most common implementation cost mentioned by participants. We mapped the mentioned implementation strategies to the ERIC framework [31]. The most frequently mentioned ERIC clusters of implementation strategies were ‘use evaluative and iterative strategies’, followed by ‘develop stakeholder interrelationships’. Implementation costs which fell outside the scope of this framework included the cost of a project manager and costs associated with workflow alterations. A project manager’s role ranged from conducting some implementation strategies to completing the administration duties to progress the project. As this role appeared to facilitate implementation, participants considered it to be an implementation cost. Participants mentioned that it was important to understand current clinical workflows and how they may be impacted from the introduction of the intervention, “*or you’re never going to get your clinicians to do anything*” *[HE]*. Consequently, the need for workflow alterations may be considered within the scope of implementation processes and costed accordingly.

Physical space was mentioned by some health economists as a potential implementation cost. Space would not be included in their costing analysis if it was not a *“big item” [IS/HE/DH]* or if *“slack is built into the system” [HE]* allowing meetings to be conducted without considerations for opportunity costs or renting the space (which would have been costed). Only one participant specifically mentioned opportunity costs for implementation. The health economist explained challenges for analysis when, *“trying to capture opportunity costs. And so the implicit question that we're always struggling to figure out is what would be the opportunity costs of someone not doing this or doing this” [HE]*. Other participants incidentally mentioned potential opportunity costs (Table 3: quote 1.4.(c)).

Participants identified costs relating to the intervention itself as non-implementation costs. In the case of digital health interventions, this included costs relating to cybersecurity and the digital backbone including hardware, software, ongoing management of a data base, and ongoing technical management.

Areas where participants found it difficult to attribute costs to implementation included existing resources because either the cost was not incurred or the additional labour to cost implementation had *“limited benefit unless there's some bigger picture” [HE/DH]*. Costing labour associated with implementation was challenging when staff had to differentiate between their implementation and clinical (or regular) duties (Table 3: quote 1.4.(d)). Intangible costs including soft skills, personal reflection time, existing relationships, level of authority, and mental load were highlighted as contributing to implementation but challenging to cost (Table 3: quote 1.4.(e)).

#### Influences on approaches for collecting implementation cost data

The results demonstrated that implementation cost estimates from data collected in practice could be influenced by several important factors. These included the wide variation in approaches to cost implementation (Table 3: quote 2.1.(a)), the burdensome nature of collection, and the availability of implementation data collection tools.

Implementation cost estimates were frequently obtained via staff time tracking methods where staff time was documented against specified activities, and salaries were applied to calculate the cost associated with each activity (Table 3: quote 2.1.(b)). Although labour intensive, this method was not seen as complex and could provide contextual insight specific to the site. However, some participants mentioned that it was at times difficult to delineate time spent in roles and responsibilities relating to the implementation activity and usual job duties. Within this approach there was a variation in practices. Detailed approaches captured all activities and personnel involved in the implementation, while other simplified approaches estimated wages only for the key personnel involved. Some project managers developed an activity template and had staff complete it prospectively with their own time allocations. Others estimated staff time and did not request staff to complete it themselves.

Other approaches to estimating implementation costs included estimating implementation costs from expert opinion (Table 3: quote 2.1.(c)), usually through experience from similar projects. Some participants expressed that the amount of available funding determined the amount of implementation costs (Table 3: quote 2.1.(d)). Economic evaluations were mentioned, although implementation costs were not frequently included in these types of analyses.

Participants discussed ways in which they retrieved informative data to value implementation cost data. Publicly available information including pay rates and awards could be used as a resource. However instead of using this resource, most participants contacted relevant teams within the organisation, for example the finance team, to obtain salaries of the personnel involved. Navigating large organisations to obtain this information was at times difficult. Most participants combined contacting relevant teams within the organisation with primary collection of information.

The collection of appropriate data to estimate implementation costs was seen as a burdensome task. This was particularly true for collectors that were not part of the implementation project team (for example clinical staff using the intervention) and when tracking staff time, as it required personnel involvement in collecting the data themselves (Table 3: quote 2.2.(a)). Suggested strategies to encourage data collection included: utilising incentives, involving collectors in the design of data collection, and building data collection into other required tasks (Table 3: quote 2.2.(b)). Achieving high accuracy and precision through frequent and comprehensive data collection was also seen as burdensome (Table 3: quote 2.3.(a)).

No standardised implementation cost data collection tools were mentioned by participants, however several attributes were considered important by participants for successful collection of implementation cost data. Participants expressed the want for practical, pragmatic, and simple tools for local implementers (Table 3: quote 2.4.(a)). A checklist-like format was suggested, as well as aligning the input with data which is already collected for another purpose (Table 3: quote 2.4.(b)). Staff time tracking was aided when implementation activities were clearly defined in advance which was commonly achieved through a purpose-built template. Participants expressed the importance of having a few clear categories for collecting the required information (Table 3: quote 2.4.(c)). Other considerations included flexibility of tools and capture formats to suit local teams (Table 3: quote 2.4.(d)), as well as ease of integration with statistical analysis software. Recording the required data digitally was favoured by participants using programs that were available and familiar, including MS Excel, RedCap and Qualtrics (Table 3: quote 2.4.(e)/(f)).

#### The value of implementation costs

Capturing implementation costs was perceived to be important for demonstrating the value of the intervention, particularly to decision makers tasked with continuing or scaling the intervention. Implementation cost estimates were used to show that the intervention was either cost saving or was justified by other benefits including improved patient experience, patience safety, and clinical outcomes (Table 3: quote 3.1.(a)). It was also suggested that implementation cost estimates can also be used to inform future scalability of the intervention (Table 3: quote 3.1.(b)). Including implementation cost estimates in grant proposals or business cases can also assist in informed financial decision making, including when to proceed with pilot implementation projects (Table 3: quote 3.1.(c)).

For some participants, the lack of research and knowledge of implementation costs within implementation science contributed to their decision to cost implementation (Table 3: quote 3.2.(a)/(b)). These participants purposefully gathered implementation cost data to address this under-researched area of implementation science. Implementation costs could be used in determining the value of implementation strategies, along with the effectiveness of implementation. While it was accepted that implementation strategies are necessary for successful implementation, costing was still important to demonstrate that funds are being used appropriately. Some funding bodies required ongoing reporting of cost spending in which implementation cost estimates were included. For others, there was no requirement to cost implementation, even though they believed it was important (Table 3: quote 3.2.(c)). Even when not a requirement, some participants would still report implementation cost estimates as part of their project management practice. Some included implementation cost estimates in disseminated reports or publication to assist others who may want to emulate the same project in their institutions.

The study design, outcomes and audience of the evaluation can impact the value of implementation costs. Implementation projects were often underpowered, with limited data available for meaningful analysis, outside of descriptive analysis. In addition, if the primary objective was not achieved, further analysis (including implementation costing) was not typically performed (Table 3: quote 3.3.(a)). Costing implementation was a challenge when funding was not available for a long enough period for rigorous evaluation or to remunerate for absent expertise including health economics (Table 3: quote 3.3.(b)). The value of evaluating implementation costs differed between audiences (Table 3: quote 3.3.(c)). It was considered important to implementation scientists and health economists. However, those with experience in implementing digital health initiatives in health services did not also share this perception (Table 3: quote 3.3.(d)).

#### Collaboration enables implementation costing

Collaboration across disciplines facilitated the overall implementation process as well as costing of implementation, even if there was a lack of expertise in implementation science within the collaboration. Some participants had not been aware of implementation science prior to starting implementation projects but had been using analogous approaches in the past (Table 3: quote 4.1.(a)). Most participants mentioned that multidisciplinary collaborations provided a rich range of perspectives which supported the implementation project. Collaboration from the beginning of a project, particularly during study design, was most beneficial and often sought out (Table 3: quote 4.2.(a)). Participants expressed that the implementation costing was aided when the required information was planned in advance with advice from health economists and those collecting the implementation cost data, typically the implementers (Table 3: quote 4.2.(b)). At times, this served a dual purpose for defining roles and responsibilities for implementers (Table 3: quote 4.3.(a)).

## Discussion

Sixteen experts in implementation science, health economics and/or digital health shared their experience of costing implementation, with examples in digital health projects. Interviewees recognised implementation costs as important and could easily identify some implementation costs (including implementation strategies, project managers and workflow alterations) and separate out non-implementation costs relating to the intervention itself. Other costs were difficult to delineate and capture in practice which was likely attributable to inconsistencies in terminology and the perceived ill-defined boundaries of implementation phases. In practice, reasons why implementation activities may not be costed include a failure to identify them, a perception they did not require reporting, or they were not considered important, all perpetuating the reported lack of awareness regarding costing implementation.

Our findings are consistent with a recent review on economic evaluations of implementation science outcomes in low- and middle-income countries which found large heterogeneity across 23 papers in how implementation resource use was conceptualised and costed [33]. Implementation strategies can be used in all phases of an implementation project [34] which likely contributes to inconsistencies in terminology and the perceived ill-defined boundaries of implementation phases that impacts capturing implementation costs in practice. Process mapping has been recommended to cost implementation and could circumvent the aforementioned issues [35, 36]. A similar approach is embedded in the cost of implementing new strategies (COINS) tool [13]. COINS maps costs to the Stages of Implementation of Completion (SIC) framework [37]. However, the level of detail required in COINS may not be suitable in some projects [14]. Simpler approaches involve summarising the project into phases and outlining activities into each phase, but are yet to be incorporated into data collection tools [10]. We found outlining phases and associated activities (Fig. 3) improved clarity when identifying potential implementation costs in our analysis. Current evidence suggests implementation costing appears to be facilitated through activity identification and categorisation to a level of detail that is required by the project.

Micro or activity-based costing approaches have been suggested for costing implementation and was reflected in our findings as the most common approach [11, 13]. These approaches have the potential to generate precise estimates [2, 38] and then may facilitate sensitivity analyses to be conducted for translating costs to other contexts by incorporating contextual differences in resource use and resource unit costs [39]. However as noted in the literature and by our participants, micro or activity-based costing can be labour intensive and burdensome [7, 8]. Data collection approaches using an onsite database (or electronic health records-base) is likely to alleviate the burden [35]. This approach may not be viable for all projects because it requires pre-existing infrastructure [35]. In our study, participants suggested that utilising data already collected for another purpose could reduce the burden. However, it is important to note that reducing the burden of data collection may lead to trade-offs in both accuracy and precision [40]. The term ‘accuracy’ refers to how close data are to their true value, while ‘precision’ is concerned with the granularity of data, which may be useful in presenting disaggregated findings or subgroup analyses. Both precision and accuracy trade-offs need to be balanced with an acceptable level of research burden on a case by case basis [39].

Micro-costing methods for collecting implementation cost data include direct observation, time-diaries/ activity logs, targeted questionnaires, key informant interviews, and onsite database (or electronic health records-base) approaches [35]. A review of micro-costing data collection tools used for health interventions outlined that the tools were developed specifically for their respective study, although some of the standardised comprehensive templates could be (and have been) generalised for public use [39]. Standardised tools promote transparency and confidence in cost estimates [39]. The use of standardised tools to cost implementation has previously been recommended [5, 7]. Participants in the current study highlighted the need for practical implementation costing tools. A standardised tool which is pragmatic, flexible and simple to collect and estimate implementation costs may improve the quality of implementation cost estimates and subsequent evaluations.

Participants described multidisciplinary collaboration as a facilitator for costing implementation. The importance of collaboration from an early stage was mentioned in our study and is consistent with previous work which recommends multi-disciplinary input during the research design phase from fields including implementation scientists and health economists [15]. Despite this knowledge, collaboration is currently lacking between implementation scientists and health economists [15]. In our study, costing implementation was hindered by the use of discipline specific terminology. For example, opportunity costs were only specifically mentioned by a health economist while other participants dismissed potential opportunity costs, unknowingly. Differences in language used between the disciplines may contribute to difficulties in collaborating and efforts should be made to develop a common understanding.

## Recommendations

We propose four recommendations, based on the findings of this study, to support the effective and efficient collection and estimation of implementation costs:

1. A set of discrete cost categories should be developed prior to the collection of implementation cost data. Ideally the categories should reflect the project/study’s implementation effort to allow for meaningful analysis from the data collected. For example, categories could reflect the implementation strategies used, in which case an implementation terminology framework like ERIC could be applied [31]. Categories could otherwise reflect resource type; for example, labour, equipment, space, supplies, and travel [9]. Other categories for cost data specific to implementation science have been suggested elsewhere [8].
2. Efforts should be made to reduce the burden of implementation cost data collection. This may be achieved through use of existing databases or leveraging data that has been collected for other purposes. However, researchers should be mindful of the accuracy and precision trade-offs that may result from these efforts.
3. Assumptions made during the implementation costing process should be reported clearly and transparently. The use of standardised checklists may assist with this. Currently there is a checklist for the conduct and reporting of cost analysis of implementation strategies which combines elements of Proctor’s report framework of D&I strategies, the CHEERS checklist for economic evaluations and Chapel and Wang’s review on cost data collection tools [9]. Another checklist is under development for conducting, reporting, and appraisal of micro-costing studies in healthcare which may also be applicable to implementation costing studies [41]. Further research to determine best practice reporting for implementation cost estimates is required.
4. Increased collaboration across disciplines, particularly between implementation scientists and health economists, is likely to promote a common understanding and facilitate implementation costing.

### Limitations

This study is limited by the data collected in the sixteen interviews conducted. In qualitative research data quality does not always improve with quantity and the sample size in this study reflects others in semi-structured interview studies which range from 5 to 25 [27]. Furthermore, data saturation and inductive thematic saturation were achieved after the third round of iterative data collection and suggested comprehensiveness of the sample. Collecting data past the point of saturation can be useful for providing rich quotes and greater levels of researcher awareness of the issues under investigation [27]. This study did not collect data past saturation.

The majority of participants were academics and therefore the results may reflect how implementation costs are estimated and data collected in academia more than frontline healthcare operations. Although, half of the academic participants were currently working in hospital systems. Exploring operational implementation outside of academic research may also provide additional insights or considerations when costing implementation. Embedding relevant experience into the research team may be useful to ensure that nuances from non-academic language are not overlooked. Due to the location of most of the participants, the results of this study will likely be most applicable to the Australian context. Stronger global representation of implementation costing practices may provide additional insights, particularly because there is no implementation costing standard in the literature.

The framework analysis method is typically used to compare between groups, however this aspect was a challenge because several participants had expertise in more than one field and/or worked across sectors. The multi- disciplinary and sectorial perspectives were valuable in this study and further highlighted the importance of collaboration. Although rigid comparisons could not be made in this study, the framework analysis technique remained useful for identifying specific perspectives.

## Conclusion

Challenges were identified in the implementation costing process, mostly relating to identifying and collecting implementation cost data. In current practice, standardised methods are not commonly used for data collection or estimation of implementation costs. Staff time tracking was the most frequently used method to cost implementation but there was variation in this approach. There is a need for pragmatic tools to facilitate the collection of implementation cost data in practice. Improved data collection practices would promote transparency and confidence in implementation cost estimates and may lead to greater reporting of implementation of costs in the literature.

## Supplementary Information

Additional file 1. COREQ checklist.

Additional file 2. Interview guide.

## Data Availability

The datasets used and/or analysed during the current study are available from the corresponding author on reasonable request.

## Abbreviations

AI: Artificial intelligence
COINS: Cost of implementing new strategies
COREQ: Consolidated Criteria for Reporting Qualitative Research Checklist
IT: Information technology
ERIC: Expert Recommendations for Implementing Change framework
