# Supplementary material for: Challenges and recommendations for collecting and quantifying implementation costs in practice: a qualitative interview study
Source: Implement Sci Commun. 2024 Oct 11;5:114. doi: 10.1186/s43058-024-00648-y (PMC11468373; doi:10.1186/s43058-024-00648-y)
Supplement: Supplementary file 1 — Additional file 1. COREQ checklist. [file 43058_2024_648_MOESM1_ESM.docx]

# Additional file 1: Consolidated criteria for reporting qualitative studies (COREQ) checklist

**Table 1**. Completed COREQ 32-item checklist for transparent and complete reporting of methods

| **COREQ checklist** | **Location where item is reported** |
| --- | --- |
| **Domain 1: Research team and reflexivity** |  |
| *Personal Characteristics* |  |
| 1. Interviewer/facilitator | Methods- Data collection |
| 2. Credentials | Methods- Data collection |
| 3. Occupation | Methods- Data collection |
| 4. Gender | Methods- Data collection |
| 5. Experience and training | Methods- Data collection |
| *Relationship with participants* |  |
| 6. Relationship established | Methods- Data collection |
| 7. Participant knowledge of the interviewer | Methods- Data collection |
| 8. Interviewer characteristics | Methods- Data collection |
| **Domain 2: study design** |  |
| *Theoretical framework* |  |
| 9. Methodological orientation and Theory | Methods- Study design |
| *Participant selection* |  |
| 10. Sampling | Methods- Study participants and recruitment |
| 11. Method of approach | Methods- Study participants and recruitment |
| 12. Sample size | Methods- Study participants and recruitment |
| 13. Non-participation | Results- Participant characteristics |
| *Setting* |  |
| 14. Setting of data collection | Methods- Data collection |
| 15. Presence of non-participants | Methods- Data collection |
| 16. Description of sample | Methods- Study participants and recruitment |
| *Data collection* |  |
| 17. Interview guide | Methods- Data collection |
| 18. Repeat interviews | Methods- Data collection |
| 19. Audio/visual recording | Methods- Data collection |
| 20. Field notes | Methods- Data collection |
| 21. Duration | Methods- Data collection |
| 22. Data saturation | Methods- Data collection |
| 23. Transcripts returned | Methods- Data collection |
| **Domain 3: analysis and findings** |  |
| *Data analysis* |  |
| 24. Number of data coders | Methods- Data analysis |
| 25. Description of the coding tree | Methods- Data analysis |
| 26. Derivation of themes | Methods- Data analysis |
| 27. Software | Methods- Data analysis |
| 28. Participant checking | Methods- Data collection |
| *Reporting* |  |
| 29. Quotations presented | Results |
| 30. Data and findings consistent | Results |
| 31. Clarity of major themes | Results, Table 3 |
| 32. Clarity of minor themes | Results, Table 3 |
