# Supplementary material for: Challenges and recommendations for collecting and quantifying implementation costs in practice: a qualitative interview study
Source: Implement Sci Commun. 2024 Oct 11;5:114. doi: 10.1186/s43058-024-00648-y (PMC11468373; doi:10.1186/s43058-024-00648-y)
Supplement: Supplementary file 2 — Additional file 2. Interview guide. [file 43058_2024_648_MOESM2_ESM.docx]

# Additional file 2: Interview Guide

# Aims and objectives

- To investigate the resource use and costs associated with the implementation of hospital-based digital health solutions.

# How to use this Guide

The first part of the guide asks questions to gain a broad overview of the system. The interviewer should then follow the questions and prompts from the section(s) of the guide. Within each section, remember:

- - 1. Not all questions need to be asked of the interviewee
    2. Should other relevant issues be raised they will be explored in the relevant section, with prompting as required
    3. Interviewers will ensure discussion progresses in a timely, yet informative manner

# Introduction

- Introduce self and the study
- Explain how interview will unfold

# General questions

## Q1. Can you tell me more about your research area?

- How long have you been working in the field?
  - If short amount of time, ask where they were beforehand?
- Do you work closely with other implementation scientists/ health economists?
  - Is your team multi-disciplinary?
- Where did you study? & What degrees?
- Have you always been an academic/ researcher?
- In your time in this role have you had the opportunity to be involved with any digital health solutions/ implementation TMFs / costing implementation?

## Q2. What comes to mind when I mention digital health innovations?

- Do you know much about it?
- How would you define it to someone else?
- When were you first exposed to digital health innovations?

## Q3. How do you use implementation science in your research (for implementation scientists)?

- How do you define implementation science?
- What is your most used TMF?
- What implementation strategies/TMF do you find most successful in hospital setting?

## Q4. Tell me what you know about implementation science or strategies (for health economists)?

- How did you apply the theories?
- In what context did you need to use it?
- Why did you need to use implementation science?

# Costs of implementation questions

## Q5. When a digital health innovation is implemented, can you tell me about any associated costs or resources that were required during the roll out?

- When were there the most resources? (start/ end or roll out)
- What/ where (department) required the most resources?
- Why were these resources brought in?
- Who chose the resources?

## Q6. Have you ever had to record/track these costs or resources? Why/ why not?

- Who recorded them?
- When did you cost (prospectively/ retrospectively)?
- What was the hardest/ easiest to cost?
- What tools were available to help with this?

## Q7. If you did want to document implementation costs/ resources used, what would be useful to achieve this?

- What tools would help you plan and budget
- Similar prompts above- who, when, why
- What tools are currently available to help with this?

## Q8. How important is it to incorporate implementation activities into a budget?

- To who is it most important?
- When would it be important

## Q9. Imagine you had a budget purely for implementation, what kinds of implementation activities would you invest in?

- Why those activities? – past experience
- For who?

# Concluding questions

## Q10. If you had unlimited budget, resources and time, how you would go about implementing a digital health solution?

- What would you do?
- How would you go about implementing it?
- What would you need to purchase?
- Do you think implementing a digital health innovation would still be an important investment? Or is there an alternative?
- How long do you think it would take to implement?

## Q11. Would you like to add anything further to our discussion?
